# Gene Delivery Therapeutics in the Treatment of Periodontitis and Peri-Implantitis: A State of the Art Review

**DOI:** 10.3390/ijms20143551

**Published:** 2019-07-20

**Authors:** Funda Goker, Lena Larsson, Massimo Del Fabbro, Farah Asa’ad

**Affiliations:** 1 Department of Biomedical, Surgical and Dental Sciences, University of Milano, 20122 Milano, Italy; 2 Department of Periodontology, Institute of Odontology, The Sahlgrenska Academy, University of Gothenburg, SE-405 30 Gothenburg, Sweden; 3 IRCCS Orthopedic Institute Galeazzi, 20161 Milano, Italy; 4 Institute of Odontology, The Sahlgrenska Academy, University of Gothenburg, SE-405 30 Gothenburg, Sweden

**Keywords:** periodontal disease, periodontal tissue regeneration, periodontal tissue engineering, peri-implant disease, gene therapy, genetic vectors

## Abstract

Background: Periodontal disease is a chronic inflammatory condition that affects supporting tissues around teeth, resulting in periodontal tissue breakdown. If left untreated, periodontal disease could have serious consequences; this condition is in fact considered as the primary cause of tooth loss. Being highly prevalent among adults, periodontal disease treatment is receiving increased attention from researchers and clinicians. When this condition occurs around dental implants, the disease is termed peri-implantitis. Periodontal regeneration aims at restoring the destroyed attachment apparatus, in order to improve tooth stability and thus reduce disease progression and subsequent periodontal tissue breakdown. Although many biomaterials have been developed to promote periodontal regeneration, they still have their own set of disadvantages. As a result, regenerative medicine has been employed in the periodontal field, not only to overcome the drawbacks of the conventional biomaterials but also to ensure more predictable regenerative outcomes with minimal complications. Regenerative medicine is considered a part of the research field called tissue engineering/regenerative medicine (TE/RM), a translational field combining cell therapy, biomaterial, biomedical engineering and genetics all with the aim to replace and restore tissues or organs to their normal function using in vitro models for in vivo regeneration. In a tissue, cells are responding to different micro-environmental cues and signaling molecules, these biological factors influence cell differentiation, migration and cell responses. A central part of TE/RM therapy is introducing drugs, genetic materials or proteins to induce specific cellular responses in the cells at the site of tissue repair in order to enhance and improve tissue regeneration. In this review, we present the state of art of gene therapy in the applications of periodontal tissue and peri-implant regeneration. Purpose: We aim herein to review the currently available methods for gene therapy, which include the utilization of viral/non-viral vectors and how they might serve as therapeutic potentials in regenerative medicine for periodontal and peri-implant tissues.

## 1. Introduction

### 1.1. Periodontitis and Peri-Implantitis

Periodontal disease is a common oral disease in humans [1]. Mostly it is characterized by a chronic inflammatory reaction of periodontal tissues that leads to the destruction of the attachment apparatus of the tooth, including alveolar bone, cementum, and the periodontal ligament (PDL) [2]. Periodontitis is usually initiated as a response to bacteria colonizing the tooth surface and gingival crevice [3]. The majority of the destruction that occurs in the host periodontal tissues is immune-mediated and inflammatory immune reactions are considered as a defense mechanism of the host to protect against infections, however, the sustained release of inflammatory cytokines leads to gingival recession and alveolar bone loss [1]. Furthermore, many factors have been suggested to contribute to the development and the severity of periodontitis, including age, smoking, diet, hormone deficiencies, bacteria, diabetes, obesity and epigenetics [4].

Similar to periodontitis, peri-implantitis is induced by most of the known periodontal pathogenic microorganisms, resulting in an inflammatory lesion in the soft tissues surrounding dental implants [5]. Since the periodontal ligament (PDL) is absent around implants, peri-implant tissues are less vascularized, and less immunologically protected, than the corresponding periodontal tissues. This is suggested to contribute to a more rapid, extensive tissue destruction observed in peri-implantitis, compared to cases of periodontitis [6].

### 1.2. Periodontal/Peri-Implant Tissue Regeneration

The fundamental goal of reconstructive periodontal/peri-implant therapy is to repair diseased supporting tissues [2,7]. At present, regeneration of damaged or diseased periodontal/peri-implant tissues mostly relies on the placement of bone grafts or bone substitutes in association with guided tissue regeneration (GTR) technique [8]. Over the past decades many biomaterials such asautografts, allografts, xenografts, cell occlusive barrier membranes, applications of autogenous and recombinant growth and differentiation factors (eg, platelet rich plasma “PRP”, recombinant human platelet-derived growth factor “rhPDGF”, recombinant human bone morphogenetic protein-2 “rhBMP-2”), and their combinations have been developed and tested to regenerate periodontal tissues with varying degrees of success [2]. Each technique and material has its own advantages thus far; the ability to regenerate completely the damaged periodontal supporting structures has not been achieved in patients [2].

### 1.3. Tissue Engineering/Regenerative Medicine (TE/RM)

Tissue engineering/regenerative medicine (TE/RM) is an established research field combining cell therapy, biomaterials, biomedical engineering and genetics all with the aim “to stimulate regeneration of tissues and organs by either implanting biomaterials for in vivo regeneration or by constructing substitutes in vitro” [9]. Mason and Dunnill (2008) described regenerative medicine as a therapy replacing or regenerating human cells, tissues, or organs to restore or establish their normal function [10]. The correct proliferation, migration, and maturation of tissue cells are dependent on coordinated interactions with soluble factors, cells, and extracellular matrix (ECM) [11]. In general terms, TE/RM mimics the natural healing process to reconstruct or regenerate damaged tissue [12].

The primary goal of regenerative periodontal bioengineering is “to promote endogenous repair mechanisms and functional regeneration through the delivery of key growth factors (GFs) or cytokines that stimulate host cells to invade a tissue defect and direct robust extra cellular matrix synthesis in vivo” [13]. Growth factors or morphogens play critical roles in regulating all the biological activities of cells, such as migration, proliferation, differentiation, maturation, and apoptosis. The introduction of recombinant human GFs and other proteins for regeneration has positively influenced the clinical outcomes of existing regenerative procedures, although the outcomes are often unpredictable, drawbacks always exist and the long-term safety data are not available with the current methods of protein delivery methods [13,14].

A major clinical challenge for TE/RM is the controlled and efficient delivery of GFs for repair of multi-tissue interfaces due to rapid degradation of the proteins [15]. The biological activity of the GFs that promote bone formation in vivo is limited by diffusion and degradation, leading to a short half-life. Several materials, including hydroxyapatite, tri-calcium phosphate, demineralized bone matrices, poly-lactic acid homo- and heterodimers, and collagen have been tested as carriers and delivery vehicles for these factors in a sustained and appropriate manner, however, appropriate local delivery still remains as a problem [16]. The main limitations of these delivery systems are inability to provide a sustained, continuous release of GFs, biodegradability, inflammatory reaction, immunological rejection, and possible transmission of disease [16]. In order to overcome these problems, alternative approaches have been established such as gene therapy applications that utilize genes encoding GF proteins to be delivered to the target cells [16]. As a summary, periodontal tissue engineering encloses three approaches: (1) Protein-based therapy (delivery of GFs), (2) cell-based therapy (delivery of stem cells), (3) gene-based therapy (delivery of genes) [12].

### 1.4. Rationale of Gene Therapy for Periodontal Tissue Engineering

Periodontal healing is dependent upon a sequence of associated events including cellular proliferation, migration, and attachment to components of the extracellular matrix as well as organic matrix synthesis and mineralization. The reconstruction of lost tissues including alveolar bone, ligament and cementum remains clinically challenging because of the involvement of three tissue types and the complexity of their relationship [17,18,19].

The goal of the tissue engineering strategies in the reconstruction of tissues in periodontal/implant region is to regenerate functional tissue through a series of key events that occur during periodontal tissue formation and growth, by means of delivering signaling molecules, cells, and scaffold/matrix to periodontal defects [12]. TE/RM has been used in the periodontal field, with an overall aim to overcome the drawbacks of the conventional biomaterials and recombinant proteins.

Basically, the general principles of healing observed in the turnover of healthy tissues can be also applied to the healing processes that take place in periodontal tissues. The cellular and molecular activities of the healing process of the lost tissues depend on the continuous interplay between growth factors and cytokines for both initiation and regulation of the process [17,18]. Current evidence demonstrated that appropriate presentation of selected multiple regulatory signals in terms of sufficient retention and optimized dose are required for effective regeneration [13]. TE/RM can be applied to enhance regeneration of the periodontal tissues, but in the case of growth factors, none of the present regenerative strategies are able to overcome the problems with protein stability or mode of delivery [13].

When applied topically, growth factors remain in the periodontal defect for a limited duration, probably due to proteolytic breakdown, receptor-mediated endocytosis, and the solubility of the delivery vehicle [19]. The main difficulties to overcome at the moment are the insufficient retention of in vitro and in vivo GF bioactivities in sustained delivery vehicles and the control of possible corresponding interactions among them when they are delivered in a single release platform [13].

Current tissue engineering approaches for therapeutic purposes typically involve one-time delivery of single growth factors, and in order to overcome the problems involved in protein-based approach, gene therapy has been developed which provides long-term exposure of multiple growth factors to the wound and maintains constant protein levels at the site of the defect [11,12,13]. In gene therapy approach, the delivery method can be tailored to the specific characteristics of the wound site [2,11,13]. The infiltrating cells can uptake the genes and continuously produce the therapeutic protein(s) in the local environment [8]. In brief, the rationale of delivery of the gene(s) encoding for therapeutic molecules for periodontal tissue engineering applications depends on its therapeutic strategy for directed, sustained and regulated protein expression [20].

## 2. Gene Therapy

### 2.1. Definition and History

A gene is a distinct sequence of nucleotides forming the chromosome portion of a cell’s DNA or chromosomal RNA. Genes are the smallest functional units of the genetic system with two main types of function: Determining the structure of the thousands of different proteins that are present in the human body and controlling where, when and in what quantity each protein is made [21].

The US Food and Drug Administration (FDA) defines gene therapy as products “that mediate their effects by transcription and/or translation of transferred genetic material and/or by integrating into the host genome and that are administered as nucleic acids, viruses, or genetically engineered microorganisms. The products may be used to modify cells in vivo or transferred to cells ex vivo prior to administration to the recipient”.

Some of the milestones in gene therapy are listed in Table 1 [22,23].

### 2.2. General Principles of Gene Therapy

The main concept of gene therapy is based on transferring of genetic information to target cells in order to alter specific genes in individual’s cells to produce a desired therapeutic effect [2,21,24,25]. In general, the selected DNA/RNA fragment/gene containing instructions for making a useful protein is packed within a vector, usually a virus, bacterium or plasmid [3,22].

### 2.3. DNA Versus RNA Delivery

Gene therapy is either viral or non-viral based on the vectors utilized for DNA delivery [3,22]. Viral gene therapy employs the inherent property of the virus to penetrate and insert DNA into the cells [3]. This viral vector acts as a vehicle and once inside the cytoplasm of the cell, it is packed into a vesicle. Later, this vesicle breaks down and releases the vector into the nucleus of the cell [22].

In the recent years, the concept of delivering mRNA (encoding therapeutic protein) has gained significant interest to overcome some drawbacks of DNA-based therapies [26]. In fact, the concept is very similar but instead of DNA, it is the RNA that encodes the target protein that is to be delivered. RNA, upon entry into cells, can get transcribed into the target proteins directly in the cytoplasm, avoiding the need for nuclear entry [26].

RNA delivery therapies have several advantages over DNA delivery, such as having no risk of insertional mutagenesis and not including complex steps. Thus, RNA delivery represents a powerful molecular means to synthesize intra-cellular proteins. Major concerns for using mRNA over DNA include its inherent instability and immunogenicity. RNA therapy requires some in vitro modifications in order to mitigate these properties before it can be used for clinical applications [26].

## 3. Delivery Vehicles: Viral Versus Non-Viral Vectors

In brief, strategies to deliver DNA can be divided into viral transduction and non-viral transfection, both of which can be performed in vivo/ex vivo [23]. Success of gene therapy mainly depends on the vectors that deliver transgenes to the target cells in an efficient manner to ensure adequate levels and duration of transgene expression [27,28].

Inadequacies of the vector are still one of this field’s key shortcomings and the selection of an appropriate vector for gene transfer depends on a number of factors, including:(1) Duration of protein expression (transient versus long term), (2) morphology of the target site, (3) route of gene delivery (ex vivo vs. in vivo), (4) target cells (dividing versus non-dividing, ease of transduction, receptor expression,) (5) desired temporal regulation of transgene expression (inducible versus constitutive), and (6) maximum threshold of vector-induced immune response acceptable for the host [2,29].

An ideal vector should have high specificity and low toxicity but there is no ideal single vector that can meet all needs for all tissues; in other words, different vectors are needed for different clinical applications [21,29].

### 3.1. Viral Vectors

Gene transfer using a virus or viral vector is known as transduction. Viruses are parasitic particles and are frequently used as the basis for vectors because of their natural ability of efficiently infecting host cells with genetic sequences [28,29]. In general, the viral genome is based on DNA or RNA, sheathed in a protein capsid, and in some cases enveloped by a phospholipid bilayer. Viral vectors, acting as delivery vehicles, are capable of prolonged transgene expression and have markedly higher transduction efficiency when compared to non-viral methods. Numerous viral vectors with different advantages/disadvantages have been studied for gene therapy including adenovirus, retrovirus, adenovirus-associated virus (AAV), lentivirus, vaccinia virus and herpes simplex virus. The main features of these systems that are most commonly used in tissue engineering are listed in Table 2 [2,24,29].

### 3.2. Non-Viral Vectors

Gene transfer using a non-viral vector is known as transfection. The main difference between viral vectors and non-viral vectors is that the latter consist of naked oligomeric DNA or plasmid DNA. The importance of the use of non-viral and safer viral vectors became clear in 1999 when the first gene-therapy-based ADASCID (adenosine deaminase: a severe combined immune deficiency) clinical trial, resulted in 4/10 patients developing leukemia. The cause was the new ADA gene being incidentally inserted into the genome close to the site of a proto-oncogene [23].

Direct injection of naked plasmid DNA is a simple and safe approach for the local delivery of the target gene to dividing and non-dividing cells [29]. Non-viral gene delivery can be classified as:

1-physical: Electroporation, sonoporation, magnetofection, electric field-induced molecular vibration, optical transfection, particle guns, micro-injection methods, lipofection;

2- chemical: Carriers such as dextrans, proteins, artificial lipids, calcium phosphate and other polymers as vectors [3,23,30].

Chemical non-viral vectors are biomaterials that can be organic (ranging from polyelectrolytes such as polylysine to liposomes or micelles from cationic surfactants) or inorganic (calcium phosphate, cadmium sulfide, carbon nanotubes, gold and iron oxide etc.). In general, these biomaterials are positively charged in order to facilitate their entry through the plasma membrane of target cells overcoming the negative charge of DNA that hinders their entrance [3]. Once inside the cell, the DNA-chemical complex is processed in the endosome and/or lysosome and the DNA is released into the cytoplasm translocating to the nucleus through the nuclear pores reaching the extra chromosomal space, where the DNA acts as local protein machinery [3].

Non-viral gene delivery is an alternative to viral gene therapy, offering enormous advantages such as: Low cost, proven safety, high DNA carrying capacity, and the possibility to produce artificial vectors according to specific needs [23]. Lipofection is a popular transfection method in cell biology and related research fields, in which vesicles of cationic lipids bind to DNA and positively charged complexes bind to the cell surface followed by uptake into the cells. Even though lipofection leads to a 5- to 100-fold increased transfection rate when compared with other chemical methods, it is not suitable for most primary isolated cells [31]. Liposome-based DNA delivery is one of the first methods to introduce exogenous DNA into eukaryotic cells, but this method is hardly used in the field of bone tissue engineering. This is possibly due to the fact that lipoplexes are cytotoxic at higher concentrations because they are able to interact with and destabilize the cell membrane [23].

The limitations of non-viral gene delivery can be listed as: Low transfection efficiency, transient expression, and non-selective cell targeting [30].

The successful transfection of cells by non-viral vectors depends on the preparation, purification, composition, size and stability of the DNA-vector complex, its solubility in the endosome and the endosomal escape to the nucleus [3,23]. Current advances in biomaterials and nanotechnology have allowed researchers to produce non-viral vectors to target specific cells with an efficacy comparable to viral vectors [31].

### 3.3. Inducible Systems for Viral Delivery

These systems typically use inducible promoters regulated by engineered and/or non-mammalian transcription factors in order to control transgene expression. The activity of these transcription factors is controlled by exogenous chemical agents such as tetracycline, which permit (‘‘on’’ state) or repress (‘‘off’’ state) transgene expression [29,32].

### 3.4. Exosomes

Exosomes are extracellular vesicles of endocytic origin secreted by cells, with a size range between 30 and 100 nm [33] that carry macromolecules including lipids, proteins, and nucleic acids (mainly RNA) [34].

Unlike other gene delivery vectors, exosomes are rapidly taken up by target cells and have a robust exosomal membrane that protects the RNA/gene of interest from digestion making them a more efficient vehicle for gene delivery [35].

There are two mechanisms by which exosomes function: (1) Secreted exosomes can selectively bind to cell surface receptors presented by recipient cells, resulting in the transduction of specific intracellular signaling and a subsequent induction of physiological changes in the recipient cells [36], (2) secreted exosomes can directly transfer intra-exosomal content such as mRNA and microRNA inside the recipient cells by fusion with cell membranes [37].

## 4. Route of Gene Delivery: Ex Vivo Versus In Vivo

**Figure 1:**
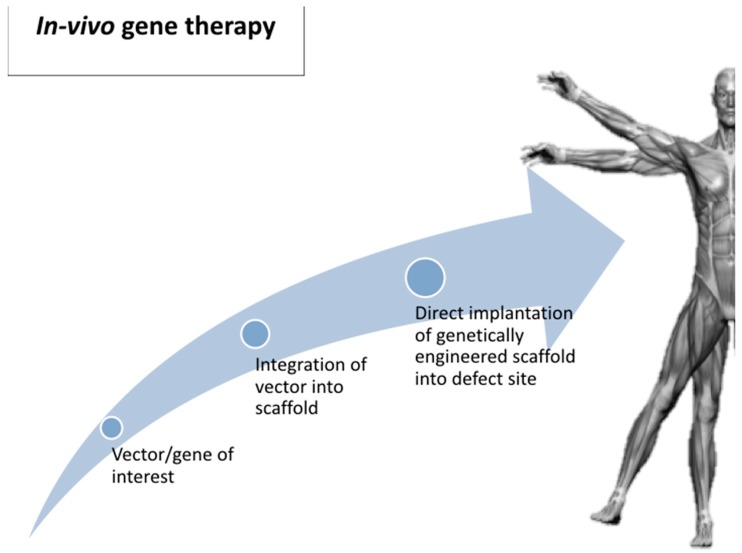
In vivo gene delivery.

In vivo gene delivery is a one-step process that may be necessary for disorders requiring immediate treatment (Figure 1). One of main challenges of this approach is low transduction efficiency and the possibility of causing inflammatory/immune response. Another limitation is the difficulty in targeting the cell population of interest [29].

**Figure 2:**
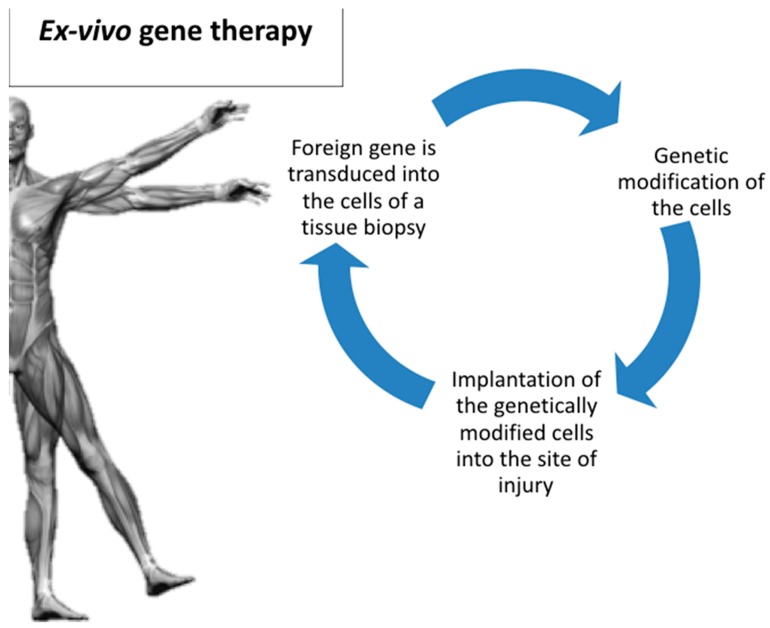
Ex vivo gene delivery.

While in ex vivo gene transfer, the foreign gene is transduced into the cells of a tissue biopsy, followed by genetic modification of these cells under in vitro conditions (Figure 2). After this preparation phase outside the body, these cells are subsequently implanted into the site of injury [21,29].

Delivery by ex vivo gene vehicle can be considered as a safer method, because cells can be screened for tumorigenicity before implantation into the host. However, these initial steps are often labor intensive, complex and involve significant cost [29].

## 5. Gene Therapy Applications for Periodontal Tissue Engineering

### 5.1. Adenovirus Vectors (AV)

Bone morphogenetic proteins (BMPs) have great potential in the regeneration of periodontal tissues. Limitations of BMP administration to periodontal lesions include their transient biological activity, and low bioavailability at the wound site. Gene transfer might serve as an alternative treatment strategy to deliver BMPs to periodontal tissues [38,39].

In 2003, Jin et al. tested adenoviral vectors for ex vivo BMP-7 and noggin gene transfer in order to stimulate tissue engineering in large mandibular bone defects in rats [38]. Noggin is an antagonist of BMP bioactivity that binds to selected BMPs and blocks the binding of BMP-2, -4, and -7 to cell surface receptors. The osseous lesions treated by AV/BMP-7 gene delivery demonstrated rapid chrondrogenesis, with subsequent osteogenesis, cementogenesis and predictable bridging of the periodontal bone defects. The results of this experiment demonstrated the first successful evidence of periodontal tissue engineering using ex vivo gene transfer of BMPs [38]. In a similar study, in 2004, the same research group tested the effect of BMP-7 and noggin gene transfer in a severe combined immunodeficient (SCID) mice model [39].

Cloned murine cementoblast cells (OCCM) were transduced using adenoviruses encoding BMP-7 and were seeded into polymer scaffolds. These 3D scaffolds were implanted into SCID mice to determine the in vivo mineral-inducing ability of the cells in mature cementoblast populations. The results of this study indicated that gene transfer of noggin inhibits biomineralization induced by cementoblasts and exogenous BMP has minimal effects on mineralization [39]. The potential of chitosan/collagen scaffold combined AV/BMP-7 was also evaluated for regeneration of the mandibular defects in dogs with beneficial results [40].

Chen et al. evaluated regeneration of the periodontal attachment apparatus in a rabbit study, using a combination of ex vivo autologous bone marrow mesenchymal stem cells (MSCs) and AV for BMP-2 gene delivery. Their results indicated that ex vivo gene transfer using stem cells as vectors may provide an advantage of slower BMP-2 release, increasing cementogenesis [8]. In a similar study, ex-vivo AV/BMP-2 gene delivery using canine periodontal ligament stem cells (PDLSCs) for regeneration of peri-implantitis defects in dogs were tested. In conclusion, ex vivo BMP2 gene delivery using PDLSCs enhanced new bone formation and re-osseointegration in peri-implantitis defects [6].

Polypeptide growth factors, such as platelet-derived growth factors (PDGFs), stimulate both cementogenesis and osteogenesis. Platelet-derived growth factors (PDGFs) also exert potent effects on wound healing including the regeneration of tooth-supporting structures. Recent advances in gene therapy offer the advantage of delivering recombinant proteins to tissues for extended periods of time in vivo [19].

In 2003, Anusaksathein et al. tested the effect of Adenovirus/PDGF-A and -B delivery to human gingival fibroblasts (HGFs) on ex vivo repair in three-dimensional collagen lattices. According to their results, PDGF gene transfer has potential for periodontal soft tissue-engineering applications [41]. A year later, the same research group published another paper on immortalized cementoblast cells (OCCM) transduced with AV for PDGF-A gene delivery in a SV40 transgenic mice model. Their findings suggested that continuous exogenous delivery of PDGF-A might delay mineral formation induced by cementoblasts, while PDGF is clearly required for mineral neogenesis [42]. The effects of gene transfer by AV/PDGF-B on human gingival fibroblasts (HGFs) and the subsequent regulation of genes and cell-surface proteins associated with cellular signaling was also evaluated in an in vitro study [43]. The findings of this study demonstrated that AV/PDGF-B gene delivery displays sustained signal transduction effects in human gingival fibroblasts [43].

Recently, the roles of the hepatocyte growth factor (HGF) associated with human dental pulp stem cells (DPSCs) in periodontal tissue regeneration were also investigated. According to the results, adenovirus-mediated transfer of hepatocyte growth factor gene to human dental pulp stem cells improved the potential for periodontal regeneration in swine model [44].

### 5.2. AAV (Adeno-Associated Viral Vectors)

Adeno-associated viral vectors are derived from a replication-deficient, non-pathogenic parvovirus with a single-stranded DNA genome and they are particularly efficient in transduction of non-dividing cells [45].

AAVs have been proposed as gene delivery vector to prevent periodontal disease progression in rats. Cirelli et al. (2009) demonstrated the use of pseudotyped adeno-associated virus vector based on serotype 1 (AAV2/1) to deliver TNF receptor-immunoglobulin Fc (TNFR:Fc) to rats subjected to *Porphyromonas gingivalis* (P.g.) [46]. The results of this study showed that AAV2/1-TNFR:Fc led to potent inhibition of periodontal disease progression [46]. In a similar study, Jiang et al. evaluated AAV-mediated RNAi knockdown (sh) of Atp6i/TIRC7 gene expression to treat periodontal disease. Mice were infected with *P. gingivalis* W50 in the maxillary periodontium to induce periodontitis. The results suggested that AAV-shRNA-Atp6i/TIRC7 therapeutic treatment might significantly improve the health of patients who suffer from *P. gingivalis*-mediated periodontal disease [47].

In a more recent in vivo experiment on mice, the role of cathepsin K (Ctsk) in chronic periodontal infection and inflammation was investigated. For this purpose, the animals were infected with *P. gingivalis* and small hairpin (sh)RNA (AAV-sh-Ctsk) was used to silence cathepsin K. According to the results, AAV-mediated silencing of Ctsk efficiently protected the animals against periodontal tissue damage and alveolar bone loss [48].

The ability of human osteoprotegerin (hOPG) recombinant adenovirus Ad5-hOPG-EGFP-transfected periodontal ligament cells (PDLCs) and BME-10X collagen membranes in combination with cell transplantation, to promote periodontal regeneration, was evaluated by Jiang et al. in 2016. The radiographic and histological results suggested that hOPG-PDLCs significantly promoted periodontal defect repair in beagle dogs. As a conclusion, this study demonstrated the potential of hOPG-modified PDLCs for periodontal tissue regeneration [49].

### 5.3. rAds (Recombinant Adeno-Viral) Vectors

The wild-type adenoviruses, including human adenovirus type 5, are associated with a number of mild disorders, such as respiratory infections. Recombinant adeno-associated virus (rAds) is a purified replication of a human parvovirus allowing it to safely be used as a gene delivery tool. rAds have been used as gene delivery vectors because of several unique features: (1) Ads have high transduction efficiency in both dividing and non-dividing cells; (2) Ads do not induce apparent phenotypic changes in transduced cells; and (3) Ads do not integrate into the host genome and remain episomal [19].

Giannobile et al. (2001) tested recombinant adenoviral vectors (rAds) encoding for the platelet-derived growth factor: A gene delivery of PDGF transgenes to cells to promote periodontal tissue regeneration. The findings demonstrated that sustained PDGF gene delivery stimulated cementoblast activity in vitro. As a conclusion, it was stated that this therapy might offer the advantage of delivering recombinant proteins to tissues for extended periods of time in vivo as a novel approach to periodontal tissue engineering [19]. In a similar in vitro experiment Zhu et al. evaluated ex-vivo-transfection of rAds (Ad2) encoding PDGF-A or PDGF-1308 to cells derived from the periodontium. As a result, it was reported that Ad2/PDGF effectively transduced cells derived from the periodontium and promoted biological activity. This study supports the potential use of gene therapy for sustained PDGF release in periodontal tissues [50].

In 2005, in vivo gene delivery using rAds encoding either the BMP-7 or the luciferase gene- using a collagen matrix was also tested. Treatment of dental implant fixtures with Ad/BMP-7 resulted in enhancement of alveolar bone defect fill, coronal new bone formation, and new bone-to-implant contact [51]. The results revealed sustained, targeted transgene expression for up to 10 days at the osteotomy sites with nearly undetectable levels by 35 days. As a conclusion, in vivo gene therapy of BMP-7 offers potential for alveolar bone engineering applications [51]. As a conclusion, results generated from these studies showed the effective transduction of the recombinant adenoviruses to the cells derived from periodontal tissues with corresponding positive effects on the modulation of DNA synthesis and cell proliferation [51].

### 5.4. Lentiviral Vectors

Lentiviral transfection was used by Xiang et al. to investigate follicular dendritic cell secreted protein (FDC-SP) on the inhibition of osteogenic differentiation of human PDL cells (hPDLCs). Their findings demonstrated that transfection with FDC-SP has a negligible adverse effect on proliferation of hPDLCs and implied the biological function of FDC-SP as a fibroblastic phenotype stabilizer by inhibiting hPDLCs differentiation into mineralized tissue-forming cells, thus regulating periodontal tissue regeneration [52].

In another in vitro experiment the role of the ephrinB2/EphB4 signaling pathway in regulating osteogenic differentiation of periodontal ligament stem cells (PDLSCs) and crosstalk between PDLSCs and pre-osteoblasts within co-culture was investigated through ephrinB2 transgenic expression in PDLSCs [53]. The human EFNB2 transgenic lentivirus PDLSCs isolated from premolar teeth were transfected with transgenic (hEfnB2-GFP-Bsd) vector or empty vector (GFP-Bsd). The data indicated that transgenic expression of ephrinB2 in PDLSCs (periodontal ligament stem cell) could promote osteogenic differentiation via stimulation of the phosphorylation of ephrinB2 and EphB4, which regulates cell communication between PDLSCs and pre-osteoblasts within the co-culture. As a result, recombinant overexpression of ephrinB2 in PDLSCs enhanced osteogenic differentiation of pre-osteoblasts [53].

Lentivirus vectors represent a potential of transducing many forms of non-dividing cells [54]. Lentiviral-based gene delivery can be an appropriate strategy for alveolar bone repair applications requiring sustained, long-term expression of therapeutic proteins but they also have some safety issues [29].

### 5.5. Non-Viral Vectors

Non-viral vectors are exciting alternatives devoid of all the drawbacks associated with viral vectors such as immunological concerns. They typically consist of plasmid DNA alone or in combination with a carrier (e.g., liposomes, cationic polymers, porous scaffolds) [29].

Elangovan et al. (2013) evaluated PDGF-B plasmids gene delivery in fibroblasts using nanosized calcium phosphate particles (NCaPP-PDGF-B) as vectors for periodontal regeneration. As a conclusion, significantly enhanced fibroblast proliferation, observed in NCaPP-PDGF-B–treated cells, confirmed the functionality of these nanoplexes [31].

In another study, transfection efficiency and toxicity of non-viral-gene-transfer methods and lentiviral vectors on human periodontal-ligament-stem-cells (hPDLSCs) were compared. The hPDLSCs were transfected by (1) Lipofectamine 2000, (2) polyethylenimine, (3) GBfectene-Elite transfection reagent, (4) X-tremeGENE HP DNA transfection reagent, and (5) magnet-assisted transfection (MATra), compared to (6) lentiviral vectors harboring a green-fluorescent-protein gene.

The transfection efficiency was measured by a fluorescence microscope and flow cytometry. Meanwhile, the cell morphology and growth status were observed to estimate the cytotoxicity. The transfection efficiency of hPDLSCs with MATra was higher than the other non-viral transfection reagents in this study, but it was far less than the lentiviral vectors [55].

### 5.6. The Gene-Activated Matrix (GAM)

The gene-activated matrix (GAM) technology is a direct gene transfer strategy, blending the two strategies—tissue engineering and local gene delivery system in periodontal tissue regeneration. GAM serves as a local bioreactor with therapeutic gene expression and provides a structural template to fill the lesion defects for cell adhesion, proliferation and synthesis of the extracellular matrix (ECM). When GAM is implanted into a tissue defect, granulation tissue fibroblasts proliferate and migrate into the GAM and take up and transiently express the therapeutic gene, thereby promoting tissue regeneration [56].

Although a number of studies focused on the safety profile of adenovirus-mediated gene therapy, few of them have addressed the local delivery of AVVs using a gene-activated matrix. Chang et al. (2009) tested local administration of AVV encoding human PDGF-B with a gene-activated collagen matrix [57]. Their results indicated that DNA vector- E1-, E3-deleted human adenovirus serotype 5 vectors are a safe method when delivered to tooth-supporting alveolar bone defects. No treatment-related toxicity or systemic involvement was found and these results support the further clinical development of AV/PDGF-B for regeneration therapy for oral and craniofacial bone applications [57].

In vitro plasmid DNA non-viral delivery, using GAM with embedded chitosan/plasmid nanoparticles encoding platelet derived growth factor (PDGF) based on porous chitosan/collagen composite scaffold was evaluated by Peng et al. in 2009 [56]. In this experiment, the chitosan/collagen scaffold acted as a three-dimensional carrier and chitosan nanoparticles condensed plasmid DNA. The plasmid DNA entrapped in the scaffolds showed a sustained and steady release over six weeks. MTT assay (colorimetric assay for assessing cell metabolic activity) demonstrated that periodontal ligament cells (PDLCs) cultured into the novel GAM achieved high proliferation. The histological results confirmed the periodontal connective tissue-like structure formation in the scaffolds after two weeks. As a result, it can be concluded that the novel GAM has potential in the application of periodontal tissue engineering [56].

In 2013, Yang et al. evaluated the effect of combining Bio-Oss (collagen bone graft material) with bone marrow stromal cells (BMSCs) transfected with the basic fibroblast growth factor (bFGF) gene-encoding plasmids on bone regeneration during mandibular distraction in rabbits [58]. According to the results of this study, bFGF promoted proliferation and differentiation of BMSCs in vitro and implantation of bFGF-expressing BMSCs combined with Bio-Oss enhanced new bone regeneration in vivo more effectively than traditional methods [58].

GAMs delivering polyplexes of polyethylenimine (PEI)-plasmid DNA (pDNA) encoding recombinant human platelet-derived growth factor-BB (rhPDGF-BB) were evaluated for promotion of periodontal wound repair in rodents [59]. Defects treated with sustained PDGF gene delivery demonstrated delayed healing coupled with sustained inflammatory cell infiltrates lateral to the osseous defects. Furthermore, significantly greater osteogenesis was observed for collagen-alone and rhPDGF-BB versus the PEI-containing groups. This non-viral gene delivery system appeared to prolong inflammatory response, slowing alveolar bone regeneration in vivo [59].

### 5.7. Bubble Liposomes and Ultrasound for Gene Delivery

Sugano et al. compared delivery of naked plasmid DNA-encoding luciferase or enhanced green fluorescent protein (EGFP) into the lower labial gingiva of Wistar rats using bubble liposomes, with or without ultrasound exposure [60]. Previously, they developed bubble liposomes as a useful carrier for gene or drug delivery, and reported that delivery efficiency was increased with high frequency ultrasound in vitro and in vivo. Hence, the aim was to examine the possibility of delivering genes into gingival tissues using bubble liposomes and ultrasound. As a conclusion, the combination of bubble liposomes and ultrasound provided an efficient technique for delivering plasmid DNA into the gingiva and bubble liposomes can be considered as a useful carrier for gene or drug delivery. This technique can be applied for the delivery of a variety of therapeutic molecules into target tissue, and in the future may serve as a useful treatment strategy for periodontitis [60].

## 6. Future Perspectives

The complexity of gene-enhanced periodontal regenerative therapeutics lies in determining which gene or gene combinations are necessary and sufficient to enhance the multiple tissue types regeneration in the periodontium. In the future, it might be possible to mimic the natural healing process by developing novel biomimetic scaffolds that react to environmental stimuli or release their cargo (proteins or genes) according to individual cellular demand. Co-operation between tissue engineers and periodontal clinicians may eventually help to overcome the challenges [13].

Scaffolds form an important component of the periodontal regenerative armamentarium [61,62] and it is expected in the near future that the use of gene-activated scaffolds, from pre-clinical studies to the clinics, will be easily accepted not only by the patients but also by the dental community, owing to its long history of acceptance. It is also expected that the cost of manufacturing such non-viral gene delivery systems will decrease, allowing treatment for a vast majority of patients [13,63].

Although gene therapy to enhance periodontal regeneration is still at a primitive stage, the constant growth in the development of non-viral vectors with better transfection efficiency suggests that DNA-based therapeutics may play a significant role in periodontal regeneration in the coming years [3].

## 7. Conclusions

The choice of vector for gene therapy depends upon a number of factors, and these parameters vary, depending upon the nature of the disease or condition to be treated. One of the main factors is the need to achieve the required level and duration of transgene expression. As an example, a genetic disease is likely to require life-long expression of a transgene, while healing of a periodontal tissue would only requires transient expression of suitable growth factors [27]. Each vector has different features, advantages and disadvantages among them. Retroviral vectors efficiently transduce dividing cells and achieve sustained and efficient transgene expression that can be propagated in target cells during mitotic division [27,29]. However, they cause insertional mutagenesis, so their clinical applications are limited just to life-threatening diseases [64].

The adenovirus vector (AV) is the most investigated viral vector in periodontal tissue regeneration procedures. AVs have highly evolved mechanisms for DNA delivery. They are not dependent on cell replication to infect cells and this leads to a high level of gene expression. AVs can infect a broad range of cell types and can be purified in high titers. They are also considered as non-oncogenic [39].

However, according to the preclinical results, AVs are inadequate for long term, stable gene expression. Another major consideration regarding using adenovirus in gene transfer procedures is the significant cytotoxic T-cell-mediated immune response that occurs when they are delivered in vivo. Studies have shown that direct systemic administration of adenoviral vectors can result in acute toxicity and hepatic pathology [28,39].

The major limitation for the use of adenoviral vectors is their high immunogenicity, which may cause inflammation, curtail transgene expression and interfere with repeated dosing [28]. When clinical translation is intended, factors such as safety, cost and intellectual property become a crucial concern for the safety of adenoviral vectors [23,29].

Second generation AVs were developed to overcome these limitations. They do not have viral proteins or genes and they are considered as less immunogenic than the first generation of AVs. Due to the lack of genes/viral proteins, second generation AVs can be generated just in the presence of helper viruses that contain the missing viral genes necessary to form a viable capsid [13].

The adeno-associated viral vector (AAV) is non-pathogenic, non-immunogenic, and able to infect a wide variety of dividing and non-dividing cells. AAVs are non-enveloped parvoviruses with the ability to transiently infect target cells, which minimizes the risk of insertional mutagenesis. For these reasons, adenoviral gene delivery has been used more extensively and safely for in vivo approaches than retroviral-based systems, but their restricted tropism and their relatively small capacity for foreign DNA have limited their use [29].

Although viral approaches have many advantages, such as prolonged transgene expression and high transduction efficiency, their perceived safety concerns poses a challenge for human translation for treatment of non-life-threatening conditions [59]. As an alternative, non-viral gene delivery systems could overcome this barrier.

These non-viral vectors may be advantageous because of their good bioavailability, low cost, and low immunogenicity due to the absence of viral proteins. They also have the ability to carry large DNA segments, and cause minimal induction of host immune responses [27,29,59]. However, non-viral vectors are far less efficient than viral vectors, although proper selection and optimization of vector-to-DNA ratio significantly enhances non-viral transfection efficiency [27,59,65].

As a conclusion, gene therapy can be considered as an alternative, which can overcome the common drawbacks of growth factors. Although current gene delivery approaches are reliable and consistent in terms of their transduction/transfection efficiency showing promising results in periodontal tissue regeneration, difficulty still exists in translating this technology to treat humans in clinics [13,26,50].

Fundamental questions that must be rigorously addressed are the selection of the suitable genes to target and employing safe vectors in order to develop a practical therapeutic modality that is both effective and safe [47]. Although gene transfer methods may circumvent many of the limitations within alternative protein delivery, current research so far provides insufficient data to permit definitive comparison [2].

## Figures and Tables

**Table 1:** Brief history of gene therapy.

**1909-** Wilhelm Johannsen introduced the term “gene”.
**1928-** Griffith performed the first experiment suggesting that bacteria are capable of transferring genetic information through a process known as “transformation”.
**1952-** Zinder and Lederberg introduced the term “transduction” as a mechanism of genetic transfer.
**1968**- Rogers and Pfuderer demonstrated a proof of concept for virus-mediated gene transfer.
**1972-** Fiedman and Roblin suggested gene therapy for genetic diseases.
**1988-** The first officially approved clinical protocol to introduce a foreign gene into humans was approved by the Recombinant DNA Advisory Committee (RAC).
**1990-** FDA approved the first time a gene therapy trial with a therapeutic attempt in humans.
**1999-** FDA restricted all clinical trials using gene therapy for nearly a decade because of the outcomes of the first gene-therapy-based clinical trial on ADASCID (adenosine deaminase: severe combined immune deficiency) as patients eventually developed leukemia (4/10).
**2003-** China became the first country to approve a gene therapy based product for clinical use.
**2012-** EMA (The European Medicines Agency) recommended for the first time a gene therapy product (Glybera) for approval in the European Union.
**December 2017-** First FDA approval of an in vivo gene-therapy product, for Luxturna from Spark Therapeutics.
**August 2018-** US National Institutes of Health advisory committee declared “gene therapies on recombinant DNA”no longer need to be reviewed before clinic studies.

**Table 2:** The main features of viral vectors.

Virus Characteristics	Retrovirus	Adenovirus	Adeno-Associated Virus	Lentivirus (HIV-1)
**Viral genome**	RNA	dsDNA	ssDNA	RNA
**Immune response**	Low	High	Moderate	Low
**Transgene expression**	Constitutitve	Transient	Constitutive/transient	Constitutive
**Immunogenicity**	Low	High	Moderate	Moderate
**Potential pathogenicity**	Low	Low	None	Low
**Insertional mutagenesis**	Yes	No	Yes/no	No

dsDNA: double stranded DNA, ssDNA: single -stranded DNA [2,29].
